# Guaranteed Income and Financial Treatment Trial (GIFT Trial or GIFTT): a 12-month, randomized controlled trial to compare the effectiveness of monthly unconditional cash transfers to treatment as usual in reducing financial toxicity in people with cancer who have low incomes

**DOI:** 10.3389/fpsyg.2023.1179320

**Published:** 2023-05-18

**Authors:** Meredith Doherty, Jonathan Heintz, Amy Leader, David Wittenburg, Yonatan Ben-Shalom, Jessica Jacoby, Amy Castro, Stacia West

**Affiliations:** ^1^School of Social Policy and Practice, University of Pennsylvania, Philadelphia, PA, United States; ^2^Sidney Kimmel Cancer Center, Thomas Jefferson University Hospital, Philadelphia, PA, United States; ^3^Mathematica, Princeton, NJ, United States; ^4^College of Social Work, University of Tennessee, Knoxville, TN, United States

**Keywords:** cancer, oncology, financial toxicity, income, unconditional cash transfers, randomized controlled trial, social determinants of health

## Abstract

Cancer-related financial hardship (i.e., financial toxicity) has been associated with anxiety and depression, greater pain and symptom burden, treatment nonadherence, and mortality. Out-of-pocket healthcare costs and lost income are primary drivers of financial toxicity, however, income loss is a pronounced risk factor for cancer patients with low incomes. There has been little progress in developing an income intervention to alleviate financial toxicity cancer patients with low incomes. Unconditional cash transfers (UCT), or guaranteed income, have produced positive health effects in experiments with general low-income populations, but have not yet been evaluated in people with cancer. The Guaranteed Income and Financial Treatment (GIFT) Trial will use a two-arm randomized controlled trial to compare the efficacy of a 12-month UCT intervention providing $1000/month to treatment as usual on financial toxicity, health-related quality of life and treatment adherence in people with cancer who have low-incomes. The study will recruit 250 Medicaid beneficiaries with advanced cancer from two comprehensive cancer centers in Philadelphia, obtain informed consent, and randomize patients to one of two conditions: (1) $1,000/month UCT or (2) treatment as usual. Both arms will receive information on financial toxicity and the contact information for their hospital social worker or financial advocate upon enrollment. Participants will complete online surveys at baseline, 3, 6, 9, and 12 months from enrollment to collect patient-reported data on primary (i.e., financial toxicity, health-related quality of life, and treatment adherence) and secondary outcomes (i.e., anxiety, depression, food insecurity, housing stability). Social security records will be used to explore the effect on mortality at 2, 3, and 5 years post-enrollment. Linear mixed-models will be used to analyze all primary and secondary continuous outcomes over time and general estimating equations with a logit link and binary distribution for all binary outcomes over time. Differences between treatment and control groups and treatment effects will be determined using models that control for age, gender, race, baseline food security, baseline housing stability, and baseline ECOG. Findings from this study will have significant implications for the development and implementation of programs and policies that address the financial burden of cancer and other serious illnesses.

## Introduction

### Background and rationale

At least one-in-three cancer patients experience cancer-related financial hardship during the course of their treatment (Yabroff et al., 2018). Cancer-related financial hardship has been associated with anxiety and depression, greater pain and symptom burden, and treatment nonadherence (Zullig et al., 2013; Delgado-Guay et al., 2015; Arastu et al., 2018). Cancer patients are at a high risk for bankruptcy, an event that has been linked to a threefold increase in the likelihood of early mortality (Ramsey et al., 2013, 2016). The adverse health effects associated with cancer-related financial hardship are called *financial* toxicity. Financial toxicity has been identified across the socioeconomic spectrum of cancer patients, but women, people of color, and low-income families experience financial hardship more often and with greater severity (Altice et al., 2016; Tucker-Seeley and Yabroff, 2016). High out-of-pocket healthcare costs and lost income are the primary drivers of financial hardship in the general cancer-affected population (Yabroff et al., 2018). However, income loss is a pronounced risk factor for low-wage workers who tend to work in sectors that lack adequate employment and income protection programs during periods of disability (Blinder et al., 2017; Blinder and Gany, 2020). As a result, cancer patients with low incomes are at greater risk of food and housing insecurity (Gany et al., 2021). Conditions of material deprivation, one domain of the social determinants of health, are robustly associated with a host of adverse health outcomes and are a critical driver of cancer health disparities (Coughlin, 2021).

The impact of financial toxicity has been well documented, however there has been little progress in developing an intervention robust enough to alleviate financial toxicity patients with cancer (Doherty et al., 2021; Offodile et al., 2022). Studies suggest that, by improving access to copayment assistance programs and optimizing insurance, financial navigation can reduce out-of-pocket costs, but treatment effects are small to moderate and programs are underutilized due to patient- and system-level factors (Shankaran et al., 2018; Yezefski et al., 2018; Monak et al., 2019; Watabayashi et al., 2020; Wheeler et al., 2020; de Moor et al., 2021; McLouth et al., 2021; Biddell et al., 2022; Smith et al., 2022). In addition to individual-level support interventions like financial navigation, experts suggest that structural, policy-level solutions are needed to mitigate the economic burden of illness in the U.S (Yabroff et al., 2020). Unconditional cash transfers (UCT), sometimes described as *guaranteed income*, have produced positive health effects in experiments with general low-income populations, but have not yet been evaluated in people with cancer who have low incomes (Gibson et al., 2018). The Guaranteed Income and Financial Treatment (GIFT) Trial will use a two-arm randomized controlled trial design to compare the effectiveness of a monthly $1,000 UCT to treatment as usual on financial toxicity, health-related quality of life and treatment adherence in people with cancer who have low incomes.

The study was funded by the One Family Foundation and the Independence Blue Cross Foundation as the Institute for Health Equity's inaugural project. Our UCT intervention stands out for its unique feature of including a waiver that allows Supplemental Security Income (SSI) recipients to receive cash assistance without jeopardizing their existing benefits. We secured this waiver through a cooperative agreement with the Social Security Administration (SSA). This waiver is necessary because cash payments count as income under the SSA rules, which can affect a recipient's eligibility for SSI benefits. SSA defines income as anything a person receives during a calendar month that can be used to fulfill their needs, whether in cash or in-kind, such as food or shelter (Social Security Administration, 2023). This waiver is a crucial component of our program as it enables SSI recipients to participate in the program without fear of losing their existing benefits. Without the waiver, our program would not be accessible to the individuals it aims to support, and its impact would be significantly limited. SSA will notify local Social Security offices that the individual is participating in an ICAP study that allows them to receive an additional $1,000 per month for 12 months.

#### Choice of comparator

In spite of growing awareness of the health effects of financial toxicity, clinical practice is widely dependent on a passive intervention model that requires patients to self-report financial and social needs (McLouth et al., 2021). Social workers and financial advocates who can help patients access copayment assistance, community grant programs, and public benefits are the standard of care. Participants in both arms of the study will receive information on financial toxicity and the contact information for their hospital social work and financial advocacy departments.

#### Research hypothesis

**Figure 1:**
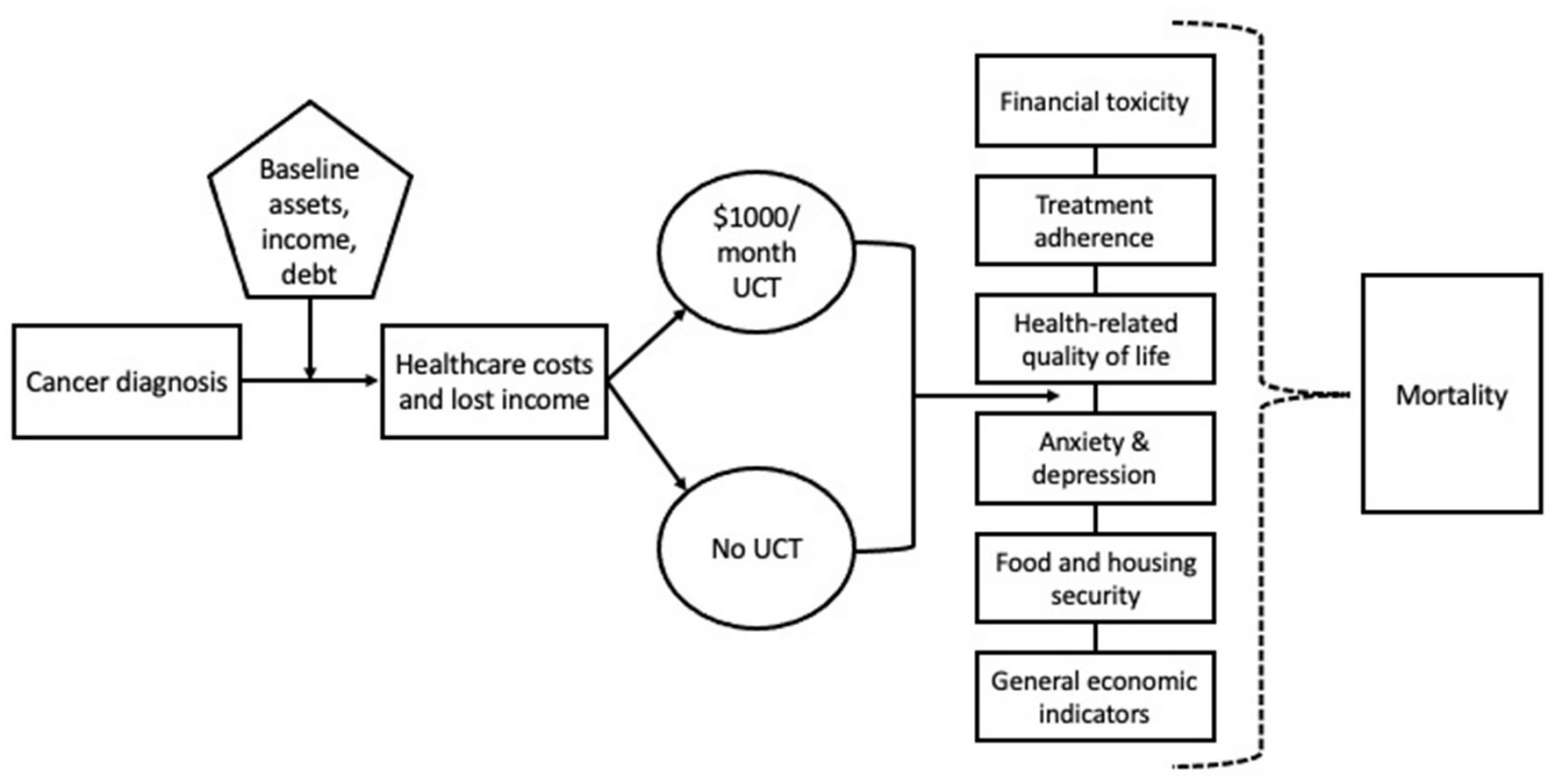
GIFT Trial causal model depicts the causal model underlying the GIFT Trial in which UCT impacts financial toxicity, health-related quality of life, treatment adherence, anxiety, depression, food, and housing instability and mortality.

A monthly UCT of $1,000 for 12 months is more effective than treatment as usual in the prevention of financial toxicity, diminished quality of life, and treatment nonadherence in people with cancer who have low incomes. See Figure 1 for the GIFT Trial causal model.

#### Study objectives

##### Primary objective

To determine if a monthly UCT of $1,000 for 12 months is more effective than treatment as usual on financial toxicity, health-related quality of life, and treatment nonadherence in people with advanced cancer (as determined by cancer stage and ECOG status) who have low income (as determined by Medicaid status).

##### Secondary objectives

To determine if monthly UCT is more effective than treatment as usual in reducing anxiety, depression, and poverty exposures (i.e., food and housing insecurity).

##### Exploratory objective

To compare the monthly UCT to treatment as usual with regard to mortality in people with cancer who have low incomes.

#### Trial design

The GIFT Trial is designed as a randomized, controlled, unblinded, multicenter superiority trial with two parallel groups and a combined primary endpoint of financial toxicity, quality of life, and treatment adherence. Randomization will be performed as block randomization with a 1:1.5 allocation stratified by age and treatment center.

## Methods

### Study setting

We selected two large, minority-serving, urban cancer centers to conduct the trial. Abramson Cancer Center at Penn Medicine and Sidney Kimmel Cancer Center at Jefferson Health.

### Eligibility criteria

Patients must provide signed (paper or electronic) informed consent before any study procedures occur. In order to verify patients' low-income status, we use Pennsylvania Medicaid beneficiary status as a proxy variable for eligibility. To be eligible for Pennsylvania Medicaid (i.e., Medical Assistance) individuals must earn <133% of the federal poverty line (FPL) or 250% FPL if considered a “disabled worker” (i.e., less than $39,900 or $75,000/year for a family of four respectively) (Pennsylvania Department of Human Services, 2023; US Centers for Medicare Medicaid Services, 2023). All participants will have household incomes under 250% FPL.

#### Inclusion criteria

Patients eligible for the trial must comply with all the following at randomization:

1. Age ≥ 18
2. Newly diagnosed or recurrent advanced cancer (Stage 3–4)
3. Receiving chemotherapy or immunotherapy (with or without radiation) at one of the recruitment sites
4. Within 12 months of receiving systemic therapy and on surveillance at one of the recruitment sites
5. ECOG performance status of 1–2
6. A Pennsylvania Medicaid beneficiary
7. A Pennsylvania resident

#### Exclusion criteria

1. Eligible for hospice (i.e., determined by provider to have a prognosis of 6 months or less) at time of randomization
2. Unable to communicate in English, Spanish, or Mandarin

### Description of study conditions

#### Intervention arm

The intervention in this trial is a philanthropically funded UCT of $1,000 per month for 12 months. Upon randomization to the intervention arm, people in the intervention arm will be contacted by the guaranteed income (GI) manager who will schedule to meet the participant either in-person at an upcoming cancer center appointment or by Zoom videoconferencing software. At the meeting, the GI manager will explain the UCT intervention in detail, explain how participation may impact public benefits, how specific public benefits will be protected during participation in the trial (i.e., Medicaid, SSI, SSDI, SNAP), and answer questions about public benefit concerns. The GI manager will then provide the participant with a USIO Inc (2023) debit card, in-person or by mail, with instructions on how to use it and the dates that they can expect to receive payment. Each month the debit card will be refilled with $1,000 and participant will receive a confirmation text message or email. Participants will be asked to provide the name and contact information of a household member or caregiver who will serve as a beneficiary for their remaining monthly UCT in the event that they enter a nursing home facility or pass away before the end of 12 months.

#### Control arm

Participants randomized to the control arm will receive an email informing them of their assignment to the control arm. We reviewed the treatment as usual practices we believe to be related to the outcomes of interest in this trial and found that all sites have at least one social worker or financial advocate that is able to help patients access routinely available financial assistance programs in the hospital and wider community, including American Cancer Society support for transportation to and from appointments and temporary lodging as needed for treatment. Participants in the treatment and control arms will be provided with information on financial toxicity and contact information for their social worker or financial advocate. They can make use of any and all financial, material, and psychosocial support programs they encounter in the course of the trial. Participants are free to engage in other clinical trials during the course of this trial.

#### Modifications

**Figure 2:**
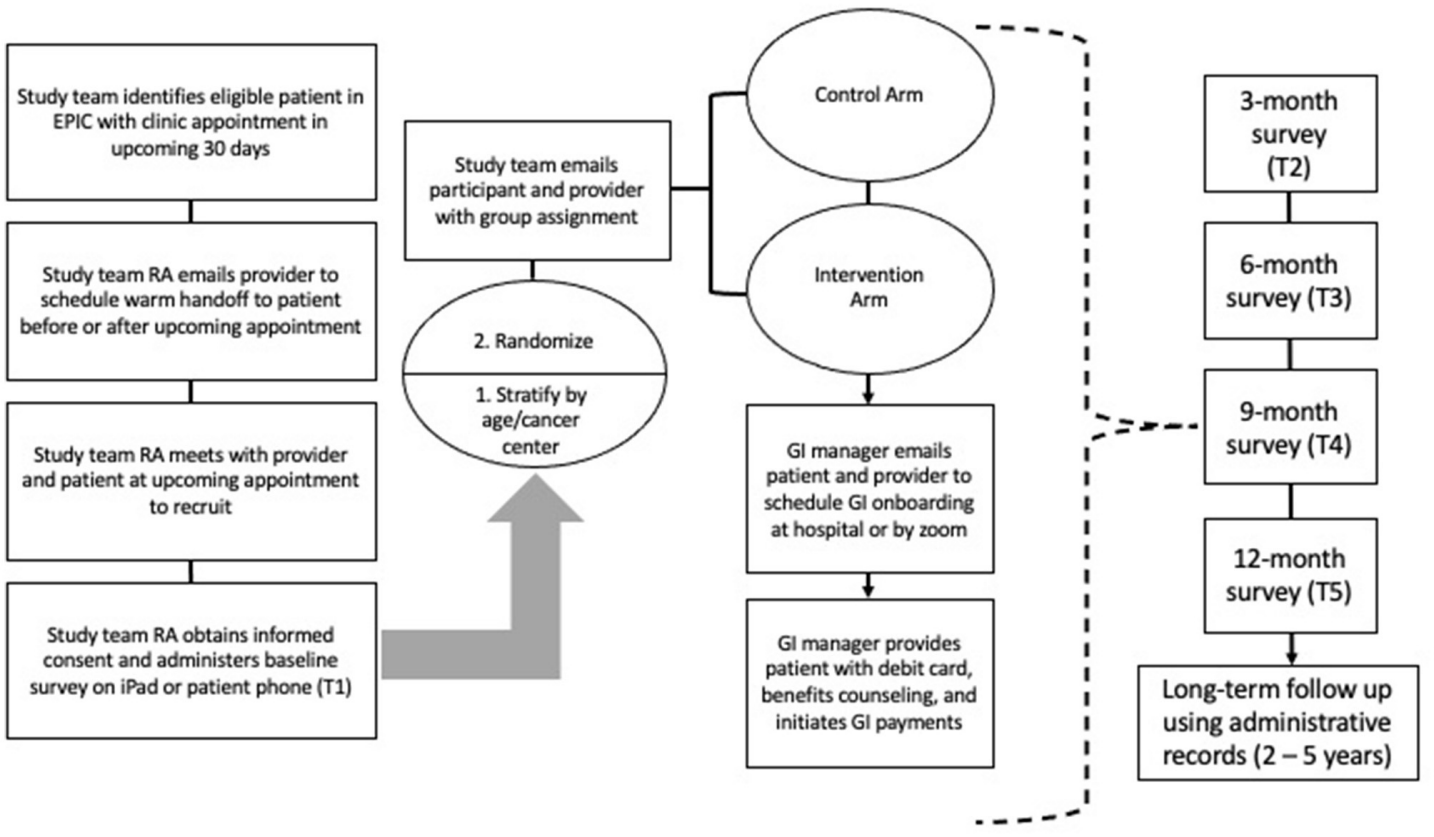
Participant flow diagram provides an overview of the study flow from recruitment through data collection. RA, Research assistant; GI, Guaranteed income.

Participants in the intervention arm may discontinue UCT payments at any time. Although we have taken every step to protect public benefits eligibility during the course of the trial there may be some interactions that we are not yet aware of, and each participant can work with the GI manager to weigh the costs and benefits of participation relative to currently unforeseen public benefits interactions. If a participant in the intervention arm dies during the 12-month intervention period, their debit card will be transferred to their designated beneficiary which is required to be next of kin or a caregiver. Having a caregiver or next of kin, however, is not required to participate in the study. In that case payments will not be redirected. If participants change cancer clinics or receive additional cancer care from another clinic, they will be able to remain in the trial. See Figure 2 for the GIFT Trial participant flow diagram.

### Outcomes

#### Primary outcome measures

The primary outcomes of interest are financial toxicity, quality of life, and treatment adherence. Primary outcomes will be measured at baseline, 3, 6, 9, and 12 months.

##### Financial toxicity

Financial toxicity will be measured using the Comprehensive Score for Financial Toxicity (COST), a validated, 11-item patient-reported outcome measure of cancer-related financial hardship that captures three domains of financial hardship (also described as *financial toxicity*): resources, affect, and financial. Scores range from 0 to 44 and lower scores indicate greater financial hardship. COST scores have been associated with quality of life, anxiety and depression (de Souza et al., 2014, 2017).

##### Health-related quality of life

Quality of life will be measured using the Rand 36–Item Short Form Health Survey (SF-36) (RAND Corp, 2022). The SF-36 is scored to produce eight subscales: physical functioning, role limitations due to physical health, role limitations due to emotional problems, energy/fatigue, emotional wellbeing, social functioning, pain, and general health. Each subscale has a score ranging from 0 to 100, produced by coding and averaging the survey responses.

##### Treatment adherence

Treatment adherence questions were developed by Gany et al. Costas-Muniz et al. (2016) to identify barriers to cancer treatment among low-income and immigrant cancer patients. The first question asks if the respondent has missed any of the following appointments in the last three months: chemotherapy/infusion, radiation, general oncology, follow-up, or any other cancer-related appointment. If the respondents indicates yes, they are asked to identify a reason for missing each of the identified appointments: did not have childcare, could not afford transportation to appointment, could not afford copayment, could not afford insurance deductible, was not covered by insurance, prior approval was not obtained, I forgot about my appointment, I was scared/anxious, or other, please describe: (free text). The second question asks if the respondent has missed a dose of cancer-related medication in the last three months and if so, select a reason why: forgot to take it, forgot to buy/pick it up, not covered by insurance, no insurance, could not afford copayment, no time to buy/pick up, do not think they will work/help, do not like the side effects, could not afford transportation to the pharmacy, other, please describe (free text). The outcome variable is binary (yes/no to having missed any appointment or medication dose in the past 12 months).

#### Secondary outcome measures

The secondary outcomes of interest are anxiety and depression, food insecurity, housing stability, and general economic indicators. Secondary outcomes will be measured at baseline, 3, 6, 9, and 12 months.

##### Anxiety and depression

Anxiety and depression will be measured with the Hospital Anxiety and Depression Scale (HADS), a 14-item scale that is considered the gold standard for measuring anxiety and depression in cancer patients. Scores range from 0 to 21 on each subscale (anxiety and depression) and higher scores indicate greater likelihood of anxiety or depression (Zigmond and Snaith, 1983; Vodermaier and Millman, 2011; The Hospital Anxiety Depression Scale, 2021).

##### Food insecurity

To measure food insecurity we will use the USDA 6-item Short Form Food Security Survey (United States Department of Agriculture, 2021).

##### Housing stability

We derived a 7-Item tool from the American Housing Survey to assess housing instability (Bureau, 2023).

##### Economic and employment variables

We will collect data on weekly hours of employment, interruptions in employment and work reductions, income sources, employment protections, essential expenses (housing, transportation, utilities, food), general impact of cancer on finances, estimated amount of personal savings and estimated amount of total credit card debt.

#### Exploratory outcome

Mortality is long-term exploratory outcome that will be measured at 2, 3, and 5 years from baseline.

##### Mortality

The intervention's potential impact on mortality will be explored using the Social Security records of all participants in the study. Mortality data from the Social Security records will be reviewed at 2, 3, and 5 years post-enrollment. The proportion of deceased participants from the intervention and control groups will be determined and compared. See Table 1 for GIFT Trial outcomes, data sources and measurement timepoints.

**Table 1:** GIFT Trial measures.

**Outcome (measure)**	**Data source**	**Baseline (T1)**	**3m (T2)**	**6m (T3)**	**9m (T4)**	**12m (T5)**	**2–5 years**
Demographic	Survey	X					
Employment, income, savings, debts, public benefits utilization		X	X	X	X	X	
Cancer type/stage		X					
Treatment nonadherence		X	X	X	X	X	
Financial toxicity (COST)		X	X	X	X	X	
Quality of life (FACT-G)		X	X	X	X	X	
Anxiety and Depression (HADS)		X	X	X	X	X	
Food insecurity (USDA)		X	X	X	X	X	
Housing instability (AHS)		X	X	X	X	X	
Mortality	Social security records						X

### Sample size

The sample size was determined by the amount of philanthropic funding we secured for the Trial which was sufficient to provide $12,000/year to 100 participants. After accounting for anticipated attrition we determined that a 250 person sample was needed – 100 in the intervention group and 150 in the control group (overrecruited for differential attrition). We conducted power analyses based on the literature and pilot study findings to determine detectable treatment effects in the primary outcomes of interest. Power analyses methods and results for each primary outcome are described below. Recruitments sites were selected for serving racially diverse, low-income patient populations. Our pilot study sample was 50% Black, 35% white, 4% Asian, 4% mixed, and 7% other. 52% of the sample identified as Hispanic or Latino.

### Recruitment

Each week the study team will use the electronic health record to view upcoming clinic appointments (in the next 2–3 weeks) and determine the study eligibility of the patients with upcoming appointments. A research assistant (RA) will contact each eligible patient's physician/nurse practitioner/physician assistant to let them know that the patient has been selected for recruitment to the study and the RA would like to meet with them prior to or after the upcoming appointment. The RA will also contact scheduling department to let them know that the patient is eligible for the study and will be approached at the upcoming appointment—both the receptionist and provider will be asked to tell the patient about the study and prepare them to be approached by the RA. The RAs will keep a participant tracking sheet and will record each step of approach and engagement. On the day of the appointment, the RA will approach the patient and use a designated quiet space to complete the informed consent (on paper) and Qualtrics baseline survey (on their phone, the RA's tablet, or with the assistance of the RA reading questions to them). The RA will provide the participant with information on financial toxicity and the contact information for their social worker or financial advocate at this time.

### Allocation

Each week participants with completed baseline surveys in Qualtrics will be randomly assigned to one of the study conditions using a computer generated 1:1.5 randomization schedule stratified by age and recruitment site. We will over recruit for the control arm to compensate for an expected 30% differential attrition rate (an estimate derived from yet unpublished U.S. guaranteed income RCTs). Randomization schedule will be stored in a password protected excel spreadsheet that can only be accessed by the principal investigator and senior research coordinator who are not involved in the day-to-day recruitment of participants.

### Data collection methods

All primary and secondary outcomes are patient-reported and will be collected using an online survey administered at baseline, 3, 6, 9, and 12 months post-enrollment. At baseline, the participants will also answer questions about their age, gender, race, education, cancer type, cancer stage, new or recurrent cancer diagnosis, household income, public benefits utilization, employment status and hours worked per week. At each data collection time point, participants will receive an email or text message with a survey link and directions for completing it. A RA will also call the participant to ask if they would like to complete the survey with them over the phone. Greenphire Clincards will be mailed to the participant's address at the 3-month data collection point and filled with $30 for each survey they complete. For the exploratory outcome of mortality, we have partnered with Social Security Administration who will provide aggregate data on participant mortality, earnings, and disability benefits at 2, 3, and 5 years from study enrollment.

#### Retention

A $30 incentive will be provided for each survey completed. Starting at each data collection time point, the study team will continue to contact the participant by email, text, and phone twice per week for 6 weeks until the survey is completed. We will call next of kin/caregiver if the survey is not completed within three weeks. If the survey is not completed within eight weeks of the data collection time point, the survey items will be treated as missing data. If response rate drops to 75% in any arm, the incentive will be increased to $50 per survey for participants in that arm of the trial.

#### Data management

Completed consent forms will be stored in a private institutional server maintained by the PIs University. Survey data will be entered and stored in Qualtrics, an online data collection and storage platform. The Qualtrics platform provides a high level of data safety and security that is HIPAA compliant for the collection and storage of personally identifiable information (Qualtrics, 2023). During periods of data analysis, data will be exported from Qualtrics to Stata SE17 as a .data file. The data will be stored in and accessed using Box, a secure cloud based platform that conforms to global compliance requirements for data privacy (Box, 2023). To protect confidentiality, we will only collect information from the participants that is essential to the study's aim of understanding the impact of UCTs on cancer patients' health and treatment outcomes. Participant's names, dates of birth, and treatment status will be collected from the electronic medical record for the purposes of outreach prior to the study. The research team will keep this information in a single, password protected excel spreadsheet stored on and accessible through a secure online document sharing platform. This information will be used to contact potential participants over the phone or at their next clinic appointment. We will maintain the contact information of all potential participants even if they decline to participate or are found ineligible in order to conduct feasibility analyses. Participants who enroll will provide identifiable personal information that will be linked to the survey data until data collection and analysis are complete. When the study is complete the data will be de-identified and stored in a secure online database with unique identifiers. The unique identifiers will be linked to the participant's personal information (name, date of birth, contact information) on a single, password protected excel spreadsheet stored on the principal investigator's password protected personal computer. Social Security Numbers (SSN) will be collected at study enrollment and entered directly into a separate, high security server maintained by the PI's University. To protect participants' eligibility for public benefits, each month the PI or Senior Research Coordinator will transfer participant SSNs and trial group allocation directly to SSA through its secure Government to Government Services Online application.

### Statistical methods

All survey data will be entered into and stored in Qualtrics, then analyzed using StataSE 17 or later, SAS v. 9.4 or later, and/or R v. 4.2.2 or later. The following descriptive statistics will be reported side-by-side for the treatment and control group at baseline: age (mean/SD and % over 64); gender (% per category); race (% per category); cancer type and stage (% per category); new or recurrent cancer diagnosis (% per category); baseline food security (mean/SD); baseline housing stability (% experience homelessness); income (mean/SD in U.S. dollars per year); employment (mean/SD in hours per week); ECOG score (% per category), COST score (mean/SD), treatment nonadherence in last 90 days (% yes). To determine if randomization was balanced Chi-square (or, if necessary due to sample size restrictions, Fisher's Exact Tests) will be used for categorical variables and independent *t*-tests (or, if non-normal, Kruskil-Wallis Tests) will be used at baseline, and between drop-outs and completers in each condition.

Linear mixed models will be used for all primary and secondary continuous outcomes over time. We will assess differences between treatment and control group after controlling for possible confounding variables. General estimating equations (GEE) with a logit link and binary distribution will be used for all binary primary and secondary outcomes over time. We will assess the odds ratio between treatment and control groups after controlling for possible confounding variables. GEEs with a log link and poisson distribution will be used for all non-binary, categorical primary and secondary outcomes over time. We will assess the risk ratio between treatment and control groups after controlling for possibly confounding variables.

Linear mixed-models and GEEs were selected because of the longitudinal nature of the study in which repeated measures will be collected across five timepoints. This approach will allow us to integrate observations from all five data collection time points into the statistical models. We plan to adjust these models by accounting for age, gender, race, new or recurrent cancer diagnosis, baseline food security, baseline housing stability, and baseline ECOG. These likely covariates were selected because they represent factors that have been associated with financial toxicity, quality of life and nonadherence in past studies (Altice et al., 2016; Yabroff et al., 2018). We suspect these variables will be correlated with the outcome variables, however, correlation structure will be determined at time of analysis.

Mortality as a long-term exploratory outcome will be analyzed at each time point separately. Chi-square (or, if necessary due to sample size restrictions, Fisher's Exact Tests) will be used to test for differences between the treatment and control groups. Logistic regressions will be used at each time point and will control for age, gender, race, new or recurrent cancer diagnosis, baseline food security, baseline housing stability, and baseline ECOG.

#### Power analysis for primary outcomes

All power calculations were conducted in Power Analysis and Sample Size (PASS) 2023. Power was calculated at alpha=0.05 with 200 patients at five timepoints. For all power estimates, we assumed an 8% mortality rate between primary timepoints; therefore, 28% of patients will be deceased by the 12 month time point (American Cancer Society, 2019). Power calculations were conducted with missing timepoints rather than on imputed data since we plan to compare imputed to non-imputed models. We further assumed an exchangeable correlation structure with a moderately strong intraclass correlation (r = 0.6).

Many estimates were obtained through the pilot analysis. We conducted an observational, pre-test/post-test pilot study in which we recruited 150 financially burdened cancer patients and provided them with a one-time grant of $1,000. We measured primary and secondary outcomes of interest prior to the intervention delivery and two months after to examine changes over time. We expect that the full trial, which will provide participants with 12 times the amount of cash that was provided in pilot, will yield higher treatment effects. We believe the estimates from the pilot are conservative estimates for the full trial, and any estimates not obtained from the pilot were overly conservative to ensure full power.

##### Financial toxicity

The pilot analysis saw a mean COST difference of 2.4 units (Treatment = 12.9, control = 10.5) and an overall standard deviation of 7.1 units. We expect the covariates we control for in the linear mixed model to have a moderate to moderately strong correlation (between 0.5 and 0.8). Although we expect to observe a larger mean COST difference in the full trial, we calculate the power of our full trial to be between 0.867 and 0.993.

##### Quality of life

The pilot analysis saw a mean SF-36 aggregate score difference of 3.4 units (Treatment = 39.8, control = 36.4) and an overall standard deviation of 29.4 units. We expect the covariates we control for in the linear mixed model to have a moderate to moderately strong correlation (between 0.5 and 0.8). Although we expect to observe a larger mean SF-36 aggregate score difference in the full trial, we calculate the power of our full trial to be between 0.1831 and 0.3294. In order to be fully powered, we will need to observe a mean SF-36 aggregate score difference of at least 6.5 assuming similar standard deviations. Although this is 2.9 units larger than observed in the pilot, we expect to see a larger difference between the treatment and control groups because of the compounding effect of monthly UCT relative to treatment as usual.

##### Treatment nonadherence

The pilot analysis saw a mean 30-day nonadherence rate of 11.5% for the control group and a mean nonadherence of 14.7% for the treatment group. We did not expect to observe an increase in nonadherence from pre to post intervention and do not have sound evidence to explain it. However, we can infer that because we only provided the pilot group with a one-time payment of $1,000, that this amount was insufficient to produce changes in 30-day nonadherence. Although we do not have sufficient evidence to corroborate nonadherence rates for our treatment group, we found the nonadherence rate for our control group to be somewhat lower than previous research on nonadherence in financially burdened cancer patients (Zullig et al., 2013; Costas-Muniz et al., 2016; Lee and Khan, 2016; Lee and Salloum, 2016; Nipp et al., 2016; Knight et al., 2018; Zhao et al., 2019). Two past studies that focused on reducing nonadherence to chemotherapy using a behavioral intervention to improve quality of life found significantly lower nonadherence to chemotherapy rates in the intervention group (19%) than in the control group (62.5%) (Cheville et al., 2015). We expect the covariates we control for in the GEE model to have a moderate to moderately strong correlation (between 0.5 and 0.8). Thus, we calculate the power to detect any difference between the treatment and control group to be between 0.065 and 0.095. If the nonadherence rate for our the trial treatment group is similar to the pilot, we will need to observe a nonadherence for the control group to be at least 32% which is possible given the nonadherence rates observed in past studies.

#### Missing data

Patterns of missing data (due to mortality and otherwise) will be analyzed. If the missing appears relatively random, multiple imputation will be used; otherwise, missingness will be addressed through last observation carried forward (LOCF). Imputed results will be compared to non-imputed results. A secondary analysis for all primary and secondary outcomes will be to test for dependence (through interactions) between treatment and time.

### Data monitoring

The PI will conduct monthly quality assurance and data integrity checks which will include checking a random set of cases in the database to ensure that key data points are available.

## Discussion

Over the last 50 years UCT demonstration projects conducted across the globe have generated a compelling body of evidence that demonstrates positive impacts on a range of health-related outcomes. UCT recipients in the US and Canada experienced improvements in birth outcomes, (Kehrer and Wolin, 1979; Chung et al., 2016) education attainment, (Maynard and Murnane, 1979; Forget, 2011) psychiatric conditions and substance abuse disorders (Costello, 2010). Globally, UCT has produced large, clinically significant reductions illness, injury, psychiatric emergencies, and related healthcare utilization (Forget, 2011, 2013; Baird et al., 2014). While other studies have demonstrated the positive health effects of UCT in other low-income populations, this trial will examine the benefit of providing ongoing income support to people with serious illnesses like cancer. Financial anxiety among cancer patients is high, but appropriate, given that 42.4 percent of U.S. cancer patients deplete their entire life's assets within two years of diagnosis (Gilligan et al., 2018). In low-income populations, income loss is a significant driver of financial toxicity, which is associated with an array of adverse health and treatment outcomes (Yabroff et al., 2018). Paid sick leave, medical leave under the Family Medical Leave Act (FMLA), and reasonable accommodations under the Americans with Disabilities Act (ADA) improve job retention in cancer patients (Blinder and Gany, 2020). However, these employment protections are either not accessible to all workers or are structured in a way that disadvantages low-wage workers (Vohra-Gupta et al., 2021). For example, just 33 percent of low-wage workers in the U.S. have any paid sick leave, compared to 95 percent of the highest paid workers (United States Bureau of Labor Statistics, 2022).

The absence of a strong social safety net in the US, relative to those of similarly developed nations, leaves many people at risk of health-related poverty (Liao et al., 2022). The consequences of poverty for individuals and society are well known, however, there are many barriers to the implementation of robust anti-poverty interventions in the U.S (Skidmore, 2018), Scientific evidence for the feasibility and effectiveness of UCT is growing, and so is bipartisan interest in guaranteed income as a cost-effective anti-poverty program (Ito, 2018). The appeal of our model is that it sidesteps one of the foremost ideological barriers to UCT: labor market participation. Although the labor market effects of UCT have been found to be negligible to positive, some are concerned that a guaranteed income would dissuade people from seeking employment (Hasdell, 2020). This study targets a population of people with serious illness, whom most people would agree should not have to work, and their family caregivers, who are engaging in the demanding work of providing care to a loved one. The US already has one federal program designed to protect income during illness and disability: Social Security Disability Insurance and Supplemental Security Income. It is well recognized that the current benefit from these programs is too low and that many recipients remain trapped in a cycle of poverty (Stapleton et al., 2006). Findings from GIFT may be directly applied to ongoing efforts to modernize disability income protections in the US. GIFT findings may have implications for non-governmental or market-based intervention as well. Health insurers and managed care organizations are interested in investing in the social needs of their beneficiaries, especially if they can demonstrate a return on investment relative to healthcare utilization and spending (Shrank et al., 2018).

The proposed study is an early effectiveness trial of UCT for cancer patients who have low incomes. Despite its many strengths there are some limitations that should be acknowledged. First, in order to verify patients' low-income status, we use Medicaid beneficiary status as a proxy variable for eligibility. As a result, this sample will not include low- to moderately low-income patients who may be experiencing financial hardship but do not have Pennsylvania Medicaid. Future studies of UCT for cancer may focus on recruiting participants with incomes slightly above the threshold for Medicaid. Second, this study relies predominantly on participants' self-reported survey data which may be limited by participant bias. In a supplemental mixed methods study, we plan to enhance GIFT trial findings by using participants' Medicaid claims data to analyze the impact of UCTs on healthcare utilization and spending and explore underlying mechanisms with qualitative interviews. Similarly, the GIFT trial will not examine the effect of UCT on caregivers, caregiver-patient dyads, or families. We may add a supplement to study these effects but have not yet developed these aims. Lastly, we are aware that what constitutes a clinically meaningful change in COST score has not been determined and that the measure has not been validated in low-income, racially diverse cancer patients. The principal investigator is currently conducting a study that aims to adapt, validate, and determine the predictive power of COST on clinical outcomes in a sample of low-income patients receiving care in a minority serving institution. These findings will aid in interpreting financial toxicity findings from the GIFT trial.

## Ethics statement

This protocol and the template informed consent forms have been approved by the University of Pennsylvania. Research assistants trained in human subjects research and the responsible conduct of research will receive additional training from the PI on the process for obtaining informed consent from participants. Informed consent forms have been audited for readability at the 6^th^ grade level. Participants will receive an information sheet and paper copy of the informed consent document for their records.

## Author contributions

MD, AC, and SW conceived of the study and are the grant holders. MD initiated and implemented the study design. YB-S, DW, AL, and JJ guide the implementation. JH provided statistical expertise in the trial design and will conduct preliminary statistical analysis. All authors contributed to the refinement of the study protocol and approved the final manuscript.

## References

[B1] Altice C. K. Banegas M. P. Tucker-Seeley R. D. Yabroff K. R. (2016). Financial hardships experienced by cancer survivors: a systematic review. J. Natl. Cancer Inst. 109, 2. 10.1093/jnci/djw205 27754926 PMC6075571

[B2] American Cancer Society (2019). Cancer Facts and Figures. Atlanta: American Cancer Society.

[B3] Arastu A. Ciminelli J. Culakova E. Lei L. Xu H. Dougherty D. W. . (2018). Association of financial toxicity (FT) with depression, anxiety, and quality of life (QoL) in older patients with advanced cancer: An analysis of 544 patients from 31 practices in the University of Rochester NCI Community Oncology Research Program (UR NCORP). Am. Soc. Clinic. Oncol. 18, 37. 10.1200/JCO.2018.36.15_suppl.e22037

[B4] Baird S. Ferreira F. H. Özler B. Woolcock M. (2014). Conditional, unconditional and everything in between: a systematic review of the effects of cash transfer programmes on schooling outcomes. J. Develop. Effectiv. 6, 1–43. 10.1080/19439342.2014.890362

[B5] Biddell C. B. Spees L. P. Petermann V. Rosenstein D. L. Manning M. Gellin M. (2022). Financial assistance processes and mechanisms in rural and nonrural oncology care settings. JCO Oncol. Pract. 18, e1392–e1406. 10.1200/OP.21.00894 35549521 PMC9509146

[B6] Blinder V. Eberle C. Patil S. Gany F. M. Bradley C. J. (2017). Women with breast cancer who work for accommodating employers more likely to retain jobs after treatment. Health Aff. (Millwood). 36, 274–281. 10.1377/hlthaff.2016.1196 28167716 PMC5559299

[B7] Blinder V. S. Gany F. M. (2020). Impact of cancer on employment. J. Clin. Oncol. 38, 302–309. 10.1200/JCO.19.01856 31804857 PMC6992498

[B8] Box (2023). Enterprise Level Data and Information Security. Available online at: https://www.box.com/security-compliance (accessed March 1, 2023).

[B9] Bureau U. C. (2023). American Housing Survey (AHS). Census.gov. Available online at: https://www.census.gov/AHS (accessed February 23, 2023).

[B10] Cheville A. L. Alberts S. R. Rummans T. A. Basford J. R. Lapid M. I. Sloan J. A. . (2015). Improving adherence to cancer treatment by addressing quality of life in patients with advanced gastrointestinal cancers. J. Pain Sympt. Manage. 50, 321–327. 10.1016/j.jpainsymman.2015.03.005 25975643 PMC5557268

[B11] Chung W. Ha H. Kim B. (2016). Money transfer and birth weight: evidence from the Alaska permanent fund dividend. Econ. Inq. 54, 576–590. 10.1111/ecin.12235

[B12] Costas-Muniz R. Leng J. Aragones A. Ramirez J. Roberts N. Mujawar M. I. . (2016). Association of socio-economic and practical unmet needs with self-reported nonadherence to cancer treatment appointments in low-income Latino and Black cancer patients. Ethn Health. 21, 118–128. 10.1080/13557858.2015.1034658 25989483 PMC4653085

[B13] Costello E. J. (2010). Family income supplements and development of psychiatric and substance use disorders among an american indian population—reply. JAMA. 304, 962–963. 10.1001/jama.2010.1241 20810370

[B14] Coughlin S. S. (2021). Social determinants of health and cancer survivorship. J. Environ. Health Sci. 7, 11–15. 34621981 PMC8494398

[B15] de Moor J. S. Mollica M. Sampson A. Adjei B. Weaver S. J. Geiger A. M. . (2021). Delivery of financial navigation services within National Cancer Institute–designated cancer centers. JNCI Cancer Spectr. 5, pkab033. 10.1093/jncics/pkab033 34222790 PMC8242138

[B16] de Souza J. A. Yap B. J. Hlubocky F. J. Wroblewski K. Ratain M. J. Cella D. . (2014). The development of a financial toxicity patient-reported outcome in cancer: The COST measure. Cancer. 120, 3245–3253. 10.1002/cncr.28814 24954526

[B17] de Souza J. A. Yap B. J. Wroblewski K. Blinder V. Araújo F. S. Hlubocky F. J. . (2017). Measuring financial toxicity as a clinically relevant patient-reported outcome: the validation of the COmprehensive Score for financial Toxicity (COST). Cancer. 123, 476–484. 10.1002/cncr.30369 27716900 PMC5298039

[B18] Delgado-Guay M. Ferrer J. Rieber A. G. Rhondali W. Tayjasanant S. Ochoa J. . (2015). Financial distress and its associations with physical and emotional symptoms and quality of life among advanced cancer patients. Oncologist. 20, 1092–1098. 10.1634/theoncologist.2015-0026 26205738 PMC4571810

[B19] Doherty M. J. Thom B. Gany F. (2021). Evidence of the feasibility and preliminary efficacy of oncology financial navigation: a scoping review. Cancer Epidemiol. Prev. Biomark. 2, 1853. 10.1158/1055-9965.EPI-20-1853 34341051 PMC9022465

[B20] Forget E. L. (2011). The town with no poverty: the health effects of a Canadian guaranteed annual income field experiment. Canadian Public Policy. 37, 283–305. 10.3138/cpp.37.3.283

[B21] Forget E. L. (2013). New questions, new data, old interventions: the health effects of a guaranteed annual income. Prev Med. 57, 925–928. 10.1016/j.ypmed.2013.05.029 23764242

[B22] Gany F. Melnic I. Ramirez J. Wu M. Li Y. Paolantonio L. . (2021). The association between housing and food insecurity among medically underserved cancer patients. Support Care Cancer. 6, 24. 10.1007/s00520-021-06254-1 34169329 PMC8225310

[B23] Gibson M. Hearty W. Craig P. (2018). Universal Basic Income A Scoping Review of Evidence on Impacts and Study Characteristics. Glasgow, Scotland: What Works Scotland.

[B24] Gilligan A. M. Alberts D. S. Roe D. J. Skrepnek G. H. (2018). Death or debt? national estimates of financial toxicity in persons with newly-diagnosed cancer. Am. J. Med. 5, 20. 10.1016/j.amjmed.2018.05.020 29906429

[B25] Hasdell R. (2020). What We Know About Universal Basic Income: A Cross-Synthesis of Reviews. Stanford, CA: Basic Income Lab.

[B26] Ito J. (2018). The paradox of universal basic ncome. Joi Itos Web. 2, 600. 10.31859/20180429.060035576235

[B27] Kehrer B. H. Wolin C. M. (1979). Impact of income maintenance on low birth weight: evidence from the Gary Experiment. J. Hum. resour. 18, 434–462. 10.2307/145316 575154

[B28] Knight T. G. Deal A. M. Dusetzina S. B. Muss H. B. Choi S. K. Bensen J. T. . (2018). Financial toxicity in adults with cancer: Adverse outcomes and noncompliance. J. Oncol. Pract. 14, e665–e673. 10.1200/JOP.18.00120 30355027

[B29] Lee M. Khan M. M. (2016). Gender differences in cost-related medication non-adherence among cancer survivors. J. Cancer Surviv. 10, 384–393. 10.1007/s11764-015-0484-5 26350680

[B30] Lee M. Salloum R. G. (2016). Racial and ethnic disparities in cost-related medication non-adherence among cancer survivors. J. Cancer Surviv. 10, 534–544. 10.1007/s11764-015-0499-y 26620816

[B31] Liao P. Zhang X. Zhang W. (2022). Endogenous health risks, poverty traps, and the roles of health insurance in poverty alleviation. Health Econ. Rev. 12, 25. 10.1186/s13561-022-00370-2 35438342 PMC9016966

[B32] Maynard R. A. Murnane R. J. (1979). The effects of a negative income tax on school performance: results of an experiment. J. Hum. Resour. 9, 463–476. 10.2307/145317

[B33] McLouth L. E. Nightingale C. L. Dressler E. V. Snavely A. C. Hudson M. F. Unger J. M. . (2021). Current practices for screening and addressing financial hardship within the NCI community oncology research Program. Cancer Epidemiol. Biomark. Prevent. 30, 669–675. 10.1158/1055-9965.EPI-20-1157 33355237 PMC8026561

[B34] Monak M. Bell K. Whitt A. (2019). Development of a financial navigation program to ease the burden of financial toxicity. J. Clin. Oncol. 37, 6565–6565. 10.1200/JCO.2019.37.15_suppl.6565

[B35] Nipp R. D. Zullig L. L. Samsa G. Peppercorn J. M. Schrag D. Taylor D. H. . (2016). Identifying cancer patients who alter care or lifestyle due to treatment-related financial distress: Coping strategies for the financial distress of cancer treatment. Psychooncology. 25, 719–725. 10.1002/pon.3911 26149817

[B36] Offodile A. C. Gallagher K. Angove R. Tucker-Seeley R. D. Balch A. Shankaran V. (2022). Financial navigation in cancer care delivery: State of the evidence, opportunities for research, and future directions. J. Clin, Oncol. 40, 2291–2294. 10.1200/JCO.21.02184 35353552

[B37] Pennsylvania Department of Human Services (2023). Medical Assistance General Eligibility Requirements. Available online at: https://www.dhs.pa.gov/Services/Assistance/Pages/MA-General-Eligibility.aspx (accessed April 7, 2023).

[B38] Qualtrics X. M. (2023). Data Protection and Privacy. Available online at: https://www.qualtrics.com/support/survey-platform/getting-started/data-protection-privacy (accessed March 1, 2023).

[B39] Ramsey S. Blough D. Kirchhoff A. Kreizenbeck K. Fedorenko C. Snell K. . (2013). Washington State cancer patients found to be at greater risk for bankruptcy than people without a cancer diagnosis. Health Aff (Millwood). 32, 1143–1152. 10.1377/hlthaff.2012.1263 23676531 PMC4240626

[B40] Ramsey S. D. Bansal A. Fedorenko C. R. Blough D. K. Overstreet K. A. Shankaran V. . (2016). Financial insolvency as a risk factor for early mortality among patients with cancer. J. Clin. Oncol. 34, 980–986. 10.1200/JCO.2015.64.6620 26811521 PMC4933128

[B41] RAND Corp (2022). 36-Item Short Form Survey from the RAND Medical Outcomes Study. Available online at: https://www.rand.org/health-care/surveys_tools/mos/36-item-short-form.html (accessed December 15, 2022).

[B42] Shankaran V. Leahy T. Steelquist J. (2018). Pilot feasibility study of an oncology financial navigation program. J. Oncol. Pract. 14, e122–e129. 10.1200/JOP.2017.024927 29272200

[B43] Shrank W. H. Keyser D. J. Lovelace J. G. (2018). Redistributing investment in health and social services: the evolving role of managed care. JAMA. 320, 2197–2198. 10.1001/jama.2018.14987 30383159

[B44] Skidmore M. J. (2018). Considering structural and ideological barriers to anti-poverty programs in the United States: an uninhibited, and unconventional, analysis. Poverty Public Policy. 10, 524–542. 10.1002/pop4.231

[B45] Smith G. L. Banegas M. P. Acquati C. Chang S. Chino F. Conti R. M. . (2022). Navigating financial toxicity in patients with cancer: a multidisciplinary management approach. CA: A Cancer J. Clinic. 72, 437–453. 10.3322/caac.21730 35584404 PMC12994614

[B46] Social Security Administration (2023). Interventional cooperative agreement program. Available online at: https://www.ssa.gov/disabilityresearch/icap.html (accessed April 6, 2023).

[B47] Stapleton D. C. O'day B. L. Livermore G. A. Imparato A. J. (2006). Dismantling the poverty trap: disability policy for the twenty-first century. Milbank Q. 84, 701–732. 10.1111/j.1468-0009.2006.00465.x 17096639 PMC2690299

[B48] The Hospital Anxiety Depression Scale (2021). Health and Quality of Life Outcomes | Full Text. Available online at: https://hqlo.biomedcentral.com/articles/10.1186/1477-7525-1-29 (accessed October 18, 2021).

[B49] Tucker-Seeley R. D. Yabroff K. R. (2016). Minimizing the “financial toxicity” associated with cancer care: advancing the research agenda. J. Natl. Cancer Inst. 108, djv410. 10.1093/jnci/djv410 26657336

[B50] United States Bureau of Labor Statistics (2022). Table 6. Selected paid leave benefits: Access. Available online at: https://www.bls.gov/news.release/ebs2.t06.htm (accessed April 26, 2022).

[B51] United States Department of Agriculture (2021). Measurement. Available online at: https://www.ers.usda.gov/topics/food-nutrition-assistance/food-security-in-the-us/measurement.aspx (accessed November 17, 2021).

[B52] US Centers for Medicare Medicaid Services (2023). Healthcare.gov. *Federal poverty level (FPL)*. Available online at: https://www.healthcare.gov/glossary/federal-poverty-level-fpl (accessed November 17, 2021).

[B53] USIO Inc (2023). Home. Available online at: https://usio.com/ (accessed February 23, 2023).

[B54] Vodermaier A. Millman R. D. (2011). Accuracy of the hospital anxiety and depression scale as a screening tool in cancer patients: a systematic review and meta-analysis. Support Care Cancer. 19, 1899. 10.1007/s00520-011-1251-4 21898134

[B55] Vohra-Gupta S. Kim Y. Cubbin C. (2021). Systemic racism and the Family Medical Leave Act (FMLA): Using critical race theory to build equitable family leave policies. J Racial Ethn Health Disparities. 8, 1482–1491. 10.1007/s40615-020-00911-7 33211249

[B56] Watabayashi K. Steelquist J. Overstreet K. A. Leahy A. Bradshaw E. Gallagher K. D. . (2020). A pilot study of a comprehensive financial navigation program in patients with cancer and caregivers. J. Natl. Compr. Cancer Netw. JNCCN. 18, 1366–1373. 10.6004/jnccn.2020.7581 33022646

[B57] Wheeler S. B. Rodriguez-O'Donnell J. Rogers C. Fulcher J. Deal A. Manning M. L. . (2020). Reducing cancer-related financial toxicity through financial navigation: Results from a pilot intervention. Cancer Epidemiol. Biomark. Prevent. 29, 694–694. 10.1158/1055-9965.EPI-20-0067

[B58] Yabroff K. R. Bradley C. Shih Y. C. T. (2020). Understanding financial hardship among cancer survivors in the United States: Strategies for prevention and mitigation. J. Clin. Oncol. 38, 292–301. 10.1200/JCO.19.01564 31804869 PMC6994250

[B59] Yabroff K. R. Zhao J. Zheng Z. Rai A. Han X. (2018). Medical financial hardship among cancer survivors in the United States: What do we know? what do we need to know? Cancer Epidemiol. Biomark. Prev. Publ. Am. Assoc. Cancer Res. Cosponsored Am. Soc. Prev. Oncol. 27, 1389–1397. 10.1158/1055-9965.EPI-18-0617 30429132

[B60] Yezefski T. Steelquist J. Watabayashi K. Sherman D. Shankaran V. (2018). Impact of trained oncology financial navigators on patient out-of-pocket spending. Am. J. Manag. Care. 24, S74–S79. 29620814

[B61] Zhao J. Zheng Z. Han X. Davidoff A. J. Banegas M. P. Rai A. . (2019). Cancer history, health insurance coverage, and cost-related medication nonadherence and medication cost-coping strategies in the United States. Value Health. 22, 762–767. 10.1016/j.jval.2019.01.015 31277821

[B62] Zigmond A. S. Snaith R. P. (1983). The hospital anxiety and depression scale. Acta. Psychiatr. Scand. 67, 361–370. 10.1111/j.1600-0447.1983.tb09716.x 6880820

[B63] Zullig L. L. Peppercorn J. M. Schrag D. Taylor Jr D. H. Lu Y. Samsa G. . (2013). Financial distress, use of cost-coping strategies, and adherence to prescription medication among patients with cancer. J Oncol Pract. 9, 60s-63s. 10.1200/JOP.2013.000971 29431038 PMC3825170

