# Corrigendum: Guaranteed Income and Financial Treatment (G.I.F.T.): a 12-month, randomized controlled trial to compare the effectiveness of monthly unconditional cash transfers to treatment as usual in reducing financial toxicity in people with cancer who have low incomes

**DOI:** 10.3389/fpsyg.2023.1245878

**Published:** 2023-07-20

**Authors:** Meredith Doherty, Jonathan Heintz, Amy Leader, David Wittenburg, Yonatan Ben-Shalom, Jessica Jacoby, Amy Castro, Stacia West

**Affiliations:** ^1^School of Social Policy and Practice, University of Pennsylvania, Philadelphia, PA, United States; ^2^Sidney Kimmel Cancer Center, Thomas Jefferson University Hospital, Philadelphia, PA, United States; ^3^Mathematica, Princeton, NJ, United States; ^4^College of Social Work, University of Tennessee, Knoxville, TN, United States

**Keywords:** cancer, oncology, financial toxicity, income, unconditional cash transfers, randomized controlled trial, social determinants of health

In the published article, an author name was incorrectly written as David Wittenberg. The correct spelling is David Wittenburg.

The authors apologize for this error and state that this does not change the scientific conclusions of the article in any way. The original article has been updated.

