# Corrigendum: Guaranteed Income and Financial Treatment Trial (GIFT Trial or GIFTT): a 12-month, randomized controlled trial to compare the effectiveness of monthly unconditional cash transfers to treatment as usual in reducing financial toxicity in people with cancer who have low incomes

**DOI:** 10.3389/fpsyg.2023.1320743

**Published:** 2023-12-13

**Authors:** Meredith Doherty, Jonathan Heintz, Amy Leader, David Wittenburg, Yonatan Ben-Shalom, Jessica Jacoby, Amy Castro, Stacia West

**Affiliations:** ^1^School of Social Policy and Practice, University of Pennsylvania, Philadelphia, PA, United States; ^2^Sidney Kimmel Cancer Center, Thomas Jefferson University Hospital, Philadelphia, PA, United States; ^3^Mathematica, Princeton, NJ, United States; ^4^College of Social Work, University of Tennessee, Knoxville, TN, United States

**Keywords:** cancer, oncology, financial toxicity, income, unconditional cash transfers, randomized controlled trial, social determinants of health

In the published article, there was an error in the article title. Instead of “Guaranteed Income and Financial Treatment (G.I.F.T.): a 12-month, randomized controlled trial to compare the effectiveness of monthly unconditional cash transfers to treatment as usual in reducing financial toxicity in people with cancer who have low incomes”, it should be “Guaranteed Income and Financial Treatment Trial (GIFT Trial or GIFTT): a 12-month, randomized controlled trial to compare the effectiveness of monthly unconditional cash transfers to treatment as usual in reducing financial toxicity in people with cancer who have low incomes”.

Additionally, in the published article, there was an error. A sentence regarding our involvement with the Social Security Administration needs to be changed on request of SSA. A correction has been made to *Introduction, Background and rationale, Paragraph 3*. This sentence previously stated:

“We collaborated with the Social Security Administration (SSA) to develop this waiver through SSA's Interventional Cooperative Agreement Program (ICAP) which provides support to competitive projects conducting interventional research on disability insurance (Social Security Administration, 2023).”

The corrected sentence appears below.

“We secured this waiver through a cooperative agreement with the Social Security Administration (SSA).”

A definite article was also used incorrectly here. Instead of “The SSA defines income as anything a person receives during a calendar month that can be used to fulfill their needs, whether in cash or in-kind, such as food or shelter (Social Security Administration, 2023).”, it should be “SSA defines income as anything a person receives during a calendar month that can be used to fulfill their needs, whether in cash or in-kind, such as food or shelter (Social Security Administration, 2023).”

The full corrected paragraph appears below.

The study was funded by the One Family Foundation and the Independence Blue Cross Foundation as the Institute for Health Equity's inaugural project. Our UCT intervention stands out for its unique feature of including a waiver that allows Supplemental Security Income (SSI) recipients to receive cash assistance without jeopardizing their existing benefits. We secured this waiver through a cooperative agreement with the Social Security Administration (SSA). This waiver is necessary because cash payments count as income under the SSA rules, which can affect a recipient's eligibility for SSI benefits. SSA defines income as anything a person receives during a calendar month that can be used to fulfill their needs, whether in cash or in-kind, such as food or shelter (Social Security Administration, 2023). This waiver is a crucial component of our program as it enables SSI recipients to participate in the program without fear of losing their existing benefits. Without the waiver, our program would not be accessible to the individuals it aims to support, and its impact would be significantly limited. SSA will notify local Social Security offices that the individual is participating in an ICAP study that allows them to receive an additional $1,000 per month for 12 months.

In addition to this, our description of SSA's GSO application was incorrect.

A correction has been made to *Methods, Data collection methods, Data Management*. The sentence previously stated: “To protect participants' eligibility for public benefits, each month the PI or Senior Research Coordinator will transfer participant SSNs and trial group allocation directly to the Social Security Administration (SSA) using an encrypted email platform developed and maintained by SSA.” The corrected sentence appears below.

“To protect participants' eligibility for public benefits, each month the PI or Senior Research Coordinator will transfer participant SSNs and trial group allocation directly to SSA through its secure Government to Government Services Online application.”

The full corrected paragraph is shown below.

Completed consent forms will be stored in a private institutional server maintained by the PIs University. Survey data will be entered and stored in Qualtrics, an online data collection and storage platform. The Qualtrics platform provides a high level of data safety and security that is HIPAA compliant for the collection and storage of personally identifiable information (Qualtrics, 2023). During periods of data analysis, data will be exported from Qualtrics to Stata SE17 as a .data file. The data will be stored in and accessed using Box, a secure cloud based platform that conforms to global compliance requirements for data privacy (Box, 2023). To protect confidentiality, we will only collect information from the participants that is essential to the study's aim of understanding the impact of UCTs on cancer patients' health and treatment outcomes. Participant's names, dates of birth, and treatment status will be collected from the electronic medical record for the purposes of outreach prior to the study. The research team will keep this information in a single, password protected excel spreadsheet stored on and accessible through a secure online document sharing platform. This information will be used to contact potential participants over the phone or at their next clinic appointment. We will maintain the contact information of all potential participants even if they decline to participate or are found ineligible in order to conduct feasibility analyses. Participants who enroll will provide identifiable personal information that will be linked to the survey data until data collection and analysis are complete. When the study is complete the data will be de-identified and stored in a secure online database with unique identifiers. The unique identifiers will be linked to the participant's personal information (name, date of birth, contact information) on a single, password protected excel spreadsheet stored on the principal investigator's password protected personal computer. Social Security Numbers (SSN) will be collected at study enrollment and entered directly into a separate, high security server maintained by the PI's University. To protect participants' eligibility for public benefits, each month the PI or Senior Research Coordinator will transfer participant SSNs and trial group allocation directly to SSA through its secure Government to Government Services Online application.

The authors apologize for these errors and state that they do not change the scientific conclusions of the article in any way. The original article has been updated.

